# A Simple Bacteremia Score for Predicting Bacteremia in Patients with Suspected Infection in the Emergency Department: A Cohort Study

**DOI:** 10.3390/jpm14010057

**Published:** 2023-12-30

**Authors:** Hyelin Han, Da Seul Kim, Minha Kim, Sejin Heo, Hansol Chang, Gun Tak Lee, Se Uk Lee, Taerim Kim, Hee Yoon, Sung Yeon Hwang, Won Chul Cha, Min Sub Sim, Ik Joon Jo, Jong Eun Park, Tae Gun Shin

**Affiliations:** 1 Department of Emergency Medicine, Samsung Medical Center, Sungkyunkwan University School of Medicine, Seoul 06355, Republic of Koreawc.cha@samsung.com (W.C.C.); minsub01.sim@samsung.com (M.S.S.); ikjoon.jo@samsung.com (I.J.J.); 2 Department of Digital Health, Samsung Advanced Institute for Health Sciences & Technology (SAIHST), Sunkyunkwan University, Seoul 06351, Republic of Korea; 3 Digital Innovation, Samsung Medical Center, Seoul 06351, Republic of Korea; 4 Department of Emergency Medicine, College of Medicine, Kangwon National University, Kangwon 20341, Republic of Korea

**Keywords:** bacteremia prediction, simple bacteremia score

## Abstract

Bacteremia is a life-threatening condition that has increased in prevalence over the past two decades. Prompt recognition of bacteremia is important; however, identification of bacteremia requires 1 to 2 days. This retrospective cohort study, conducted from 10 November 2014 to November 2019, among patients with suspected infection who visited the emergency department (ED), aimed to develop and validate a simple tool for predicting bacteremia. The study population was randomly divided into derivation and development cohorts. Predictors of bacteremia based on the literature and logistic regression were assessed. A weighted value was assigned to predictors to develop a prediction model for bacteremia using the derivation cohort; discrimination was then assessed using the area under the receiver operating characteristic curve (AUC). Among the 22,519 patients enrolled, 18,015 were assigned to the derivation group and 4504 to the validation group. Sixteen candidate variables were selected, and all sixteen were used as significant predictors of bacteremia (model 1). Among the sixteen variables, the top five with higher odds ratio, including procalcitonin, neutrophil–lymphocyte ratio (NLR), lactate level, platelet count, and body temperature, were used for the simple bacteremia score (model 2). The proportion of bacteremia increased according to the simple bacteremia score in both cohorts. The AUC for model 1 was 0.805 (95% confidence interval [CI] 0.785–0.824) and model 2 was 0.791 (95% CI 0.772–0.810). The simple bacteremia prediction score using only five variables demonstrated a comparable performance with the model including sixteen variables using all laboratory results and vital signs. This simple score is useful for predicting bacteremia-assisted clinical decisions.

## 1. Introduction

Bacteremia is a major cause of morbidity and requires early detection and appropriate antibiotics [1,2,3]. Blood culture sampling is a mandatory method used to detect bacteremia and it is commonly performed for various patients from less severe infection to septic shock in emergency departments [4,5]. However, the prevalence of bacteremia is 7–20%, with a high rate of false positives, and the indication(s) for performing blood cultures is not well established, and thus remains controversial [6,7]. This results in unnecessary invasive procedures, consumption of resources, increased costs, inappropriate or delayed use of antibiotics, and prolonged hospital admission [8,9]. The rate of false positives in blood cultures or contamination is often the highest in emergency departments (EDs). Robertson et al. reported contamination rates of 11.7% in the ED versus 2.5% in other hospital areas [10]. 

Several clinical tools have been developed to predict bacteremia using biomarkers and clinical scores [11]. Consequently, prediction tools that enable exclusion of bacteremia are highly desirable to increase the cost effectiveness of microbiological tests [12]. Shapiro et al. suggested indications for blood culture if at least one major or two minor criteria were present among 13 clinical parameters associated with high risk; otherwise, patients are classified as “low risk” and unnecessary blood cultures may be omitted [13,14]. In addition to clinical findings, many studies have suggested that laboratory investigations, such as procalcitonin (PCT) and neutrophil–lymphocyte ratio (NLR), may play a useful role in predicting bacteremia [15,16]. 

Although many efforts have been made to predict bacteremia, there are no detailed guidelines specifying which patients should undergo blood culture testing, and no simple prediction score for bacteremia has yet been developed. To identify patient groups at low risk for bacteremia and optimize the blood culture practice, we aimed to develop a simple scoring system that has a discriminatory value for predicting bacteremia and can help physicians classify bacteremia risk. 

## 2. Materials and Methods

This large retrospective cohort study was conducted at the Samsung Medical Center, a university-affiliated, tertiary care referral hospital, located in Seoul, South Korea, from 10 November 2014 to 10 November 2019. This study was approved by the Institutional Review Board (IRB, 2023-09-144) of Samsung Medical Center. Given the retrospective nature of the study and the use of anonymized patient data, requirements for informed consent were waived. The study population comprised patients >18 years of age with suspected infection who underwent blood culture sampling and administration of antibiotics at ED admission, excluding those with septic shock [17]. 

### 2.1. Study Design 

The primary goal was to develop a simple bacteremia score (model 2) and to compare its predictive accuracy with a reference model (model 1). 

Data were retrospectively collected from electronic medical records. The population was randomly divided into a derivation cohort (80% of randomly selected samples) and a validation cohort (20% of randomly selected samples) with R statistical programming. The sampling code splits 80% of data selected randomly into the training set and the remaining 20% of samples into the test dataset. The sampling function in R randomly picks 80% of rows from the dataset without replacement. Sixteen candidate variables possibly associated with bacteremia were analyzed, including epidemiological factors, vital signs, and laboratory results, using simple comparison and univariable and multivariable logistic regression analyses of the derivation cohort to identify risk factors using variables with a *p* value < 0.05. The cut-off values for each variable were determined using the area under the receiver operating characteristic (ROC) curve (AUC) and based on a literature review. A reference model (model 1) was developed using the derivation cohort. Model 1 comprised variables that were found to be risk factors for bacteremia in multivariable logistic regression. Among the variables used in model 1, the top 5 with the highest odds ratio (OR) were selected to develop a simple bacteremia score model (model 2) using a regression coefficient-based scoring method. Predictive factors for bacteremia were identified using multivariable analysis and were assigned a weighted value to each factor using β coefficients that reflect predictive power. The β coefficients were rounded to the nearest whole number. A rounded number for each predictive factor was assigned to the bacteremia score. The overall risk score was calculated as the sum of these scores. 

Finally, an ROC curve was generated, and the AUC was used to calculate the performance of the prediction model using the validation cohort. The prediction performances of the two models were compared along with 1 variable (PCT) that exhibited the most potent association with bacteremia. 

### 2.2. Statistical Analysis 

Standard descriptive statistics were used for all variables including baseline demographics and outcomes. The results are expressed as the median and interquartile range (IQR) for continuous variables and as the number with percentage for categorical data. Continuous variables were compared using the Wilcoxon rank-sum test. Categorical variables were compared using the chi-squared test. Univariate and multivariate logistic regression analyses were performed to assess variables related to bacteremia. Multivariate analysis using the logistic regression model was used to evaluate independent predictors of bacteremia, as measured by the estimated OR with corresponding 95% confidence interval (CI). Adjusted variables were selected based on their clinical relevance to bacteremia, and significant associations in the univariate analysis were entered into a stepwise logistic regression model. In the stepwise logistic regression model, the *p* value threshold to enter into the model was set at 0.25, and at 0.1 to be excluded from the model. The goodness-of-fit of the final logistic regression model was assessed using the Hosmer–Lemeshow test. The variables entered into the model were assigned a score based on the ORs to calculate a simple and easy clinical prediction scale. The discrimination performance of the risk index was assessed using the AUC, and the optimum cut-off value was chosen for optimal sensitivity and specificity. DeLong’s test was used to compare ROC curves between the models. Differences with *p* < 0.05 were considered to be statistically significant. Statistical analysis was performed using Stata version 18.0 (StataCorp LLC, College Station, TX, USA). 

### 2.3. Definition 

The cut-off values for predictors were as follows: age > 65 years; systolic blood pressure (SBP) < 90 mmHg or mean arterial pressure (MAP) ≤ 65 mmHg; heart rate (HR) > 130 beats/min; respiratory rate (RR) ≥ 22 breaths/min; body temperature (BT) < 36 °C or >38 °C; white blood cell (WBC) count < 4000 or >12,000 cells per microliter; platelet count (PLT) < 150,000 cells per microliter; band neutrophil > 5%, absolute neutrophil count (ANC) < 1.5 or >8.3 cells per microliter; absolute lymphocyte count (ALC) > 2.9 or <0.9 cells per microliter; neutrophil–lymphocyte ratio (NLR, [ANC/ALC]) ≥ 10 determined based on previous studies. Five “remnant” variables, including albumin < 3.5 g/dL, creatinine > 1.5 mg/dL, lactate (Lac) > 2 mmol/L [16], C-reactive protein (CRP) > 8 mg/dL, and PCT > 0.5 ng/mL, were determined by the AUC. The outcome variables were bacteremia and positive blood culture results. A positive blood culture was defined as the growth of pathogens compatible with the clinical presentation. Common skin flora (e.g., coagulase-negative staphylococci [CNS], *Corynebacterium* spp., *Cutibacterium* spp., and *Bacillus* spp.) were regarded as contamination and defined as a false-positive blood culture. Two independent physicians assessed “true” blood culture positivity.

## 3. Results

### 3.1. Baseline Characteristics

**Figure 1:**
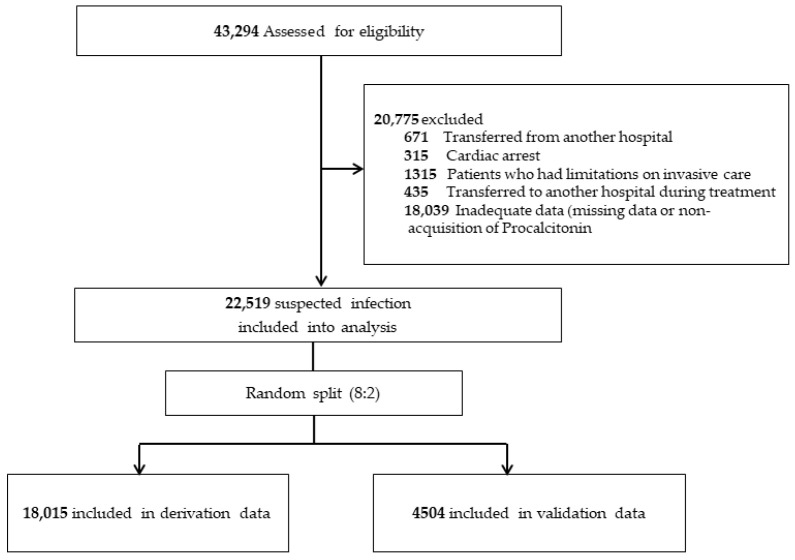
Study flow.

A total of 43,294 patients from the institution registry were assessed for eligibility during the study period. Of these, we excluded a total of 20,775 patients who transferred from another hospital or to another hospital at the ED, who presented with cardiac arrest, who had limitations on invasive care, who had inadequate data such as missing data or non-acquisition of PCT. Ultimately, 22,519 patients were included in the analysis, of whom 18,015 (80%) were randomly assigned to the derivation cohort and 4504 (20%) to the validation cohort (Figure 1). 

**Figure 2:**
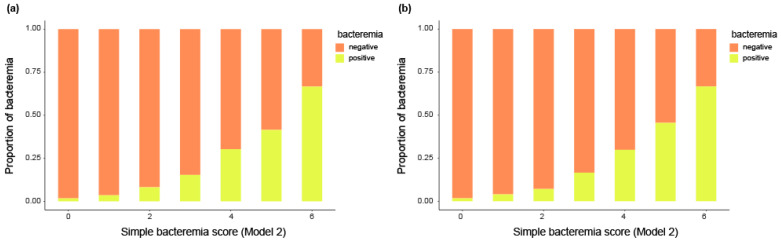
Distribution of bacteremia according to the simple bacteremia score levels in the derivation and validation cohort. (**a**) Derivation cohort. (**b**) Validation cohort.

Among the 22,519 patients, 12,232 (54.3%) were male and 9741 (43.3%) were >65 years of age. Additionally, among the 22,519 patients, 174 (0.8%) were contaminated (false positive) and 2701 (12.0%) had bacteremia (true positive). The baseline characteristics of all patients are summarized in Table 1. The total incidence of bacteremia was 12% (n = 2701/22,519), while the proportion of Gram-positive bacteremia (GPB) was 2.8% (n = 693/22,519) and Gram-negative bacteremia (GNB) was 8.6% (n = 1941/22,519). The incidence of bacteremia was 11.9% (n = 2145) in the derivation cohort and 12.3% (n = 556) in the validation cohort. The proportion of GPB was 2.7% (n = 491) and GNB was 8.6% (n = 1551) in the derivation cohort, while the proportion of GPB was 2.9% (n = 132) and GNB was 8.7% (n = 390) in the validation cohort (Table S1). The proportion of bacteremia increased according to the simple bacteremia score in both cohorts (Figure 2). 

### 3.2. Predictors of Bacteremia and Developing a Reference Model (Model 1) 

In the analysis comparing the no-bacteremia and bacteremia cohorts, 16 variables were used for multivariable logistic regression analysis. The 16 variables included age > 65 years, SBP < 90 mmHg or MAP ≤ 65 mmHg, HR > 130 beats/min, RR ≥ 22 breaths/min, BT < 36 °C or >38 °C, WBC < 4000 or >12,000 cells per microliter, PLT < 150,000 cells per microliter, band neutrophil > 5%, ANC < 1.5 or >8.3, ALC > 2.9 or <0.9, NLR ≥ 10, albumin < 3.5 g/dL, Creatinine > 1.5 mg/dL, Lac > 2 mmol/L, CRP > 8 mg/dL, and PCT > 0.5 ng/mL. Consequently, all 16 variables were significantly associated with bacteremia in the univariate analysis (all *p* < 0.001) (Supplementary Table S2). This association remained consistently significant after adjusting for confounding factors in the multivariate logistic regression model (Supplementary Table S3). A reference model (model 1) was developed using the derivation cohort. 

### 3.3. Score Development and Developing a Simple Bacteremia Score (Model 2)

Among the sixteen variables, PCT, NLR, Lac, PLT, and BT were the top five variables associated with bacteremia in the derivation cohort. Subsequently, a final logistic regression was performed to develop a simple bacteremia score (model 2) with the top five variables in the derivation cohort (Table 2). More specifically, these top five variables were significantly associated with an increased risk for bacteremia in a multivariable logistic regression: PCT (adjusted odds ratio [aOR] 4.65 [95% confidence interval (CI) 4.16–5.20]; *p* < 0.001); NLR (aOR 2.27 [95% CI 2.05–2.51]; *p* < 0.001); Lac (aOR 2.00 [95% CI 1.81–2.20]; *p* < 0.001); PLT (aOR 2.72 [95% CI 2.15–3.43]; *p* < 0.001); BT (aOR 2.04 [95% CI 1.85–2.25]; *p* < 0.001). The Hosmer–Lemeshow test revealed a goodness-of-fit of 11.18 (*p* = 0.131). 

Using these top five variables, a simple score was developed. To simplify the assessment of bacteremia risk, we used points-based scoring systems, which enable a rapid decision for risk without the use of computers or electronic devices. To develop point-based scoring systems, the OR (β coefficients) of this model were converted into integer single risk scores by rounding to the nearest whole number. The points associated with each level of each risk factor are defined relative to the points associated with an increase in a specified continuous variable. The calculated points were assigned as independent variables. The simple bacteremia score was developed by summing the computed component variables; the total score ranged from 0 to 6 points (Table 3). 

### 3.4. Bacteremia Rate According to Score

**Figure 3:**
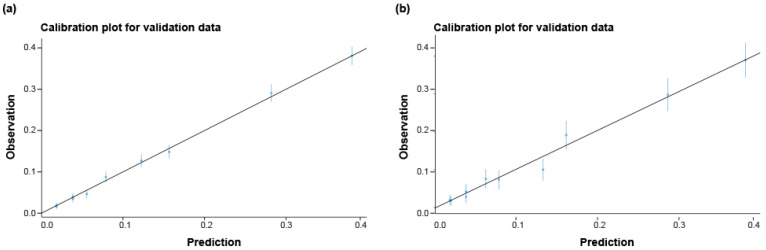
Calibration of simple bacteremia score in the derivation and validation cohort. Calibration plot indicating the agreement between model predictions (predicted probabilities) and observed frequencies. Individual data points are shown for the derivation and validation cohort. (**a**) Derivation cohort. (**b**) Validation cohort.

The rate of bacteremia according to the assigned scores is presented in Figure 3. In the derivation cohort, the rate of bacteremia gradually increased with the simple bacteremia scores: 1.8% at score 0; 3.5% at score 1; 8.2% at score 2; 15.4% at score 3; 30.3% at score 4; 41.5% at score 5; 66.7% at score 6 (*p* < 0.01). This trend in the prevalence of bacteremia was similar in the validation cohort: 1.7% at score 0; 4.0% at score 1; 7.1% at score 2; 16.6% at score 3; 29.9% at score 4; 45.5% at score 5; 66.7% at score 6 (*p* < 0.01).

### 3.5. Validation

**Figure 4:**
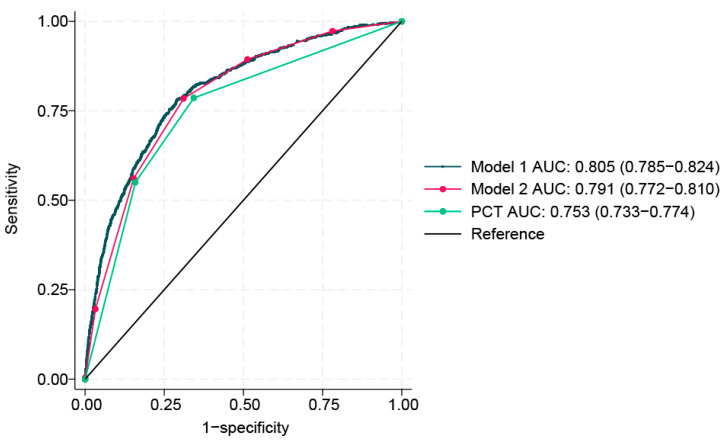
Receiver operating characteristic (ROC) curves of model 1, simple score, and procalcitonin to predict bacteremia in the validation data.

Prediction models were proposed and the performance of each was assessed using ROC curves and calibration plots. In the derivation cohort, the AUC for predicting bacteremia in model 1 was calculated to be 0.803 (95% CI 0.794–0.813), model 2 was 0.790 (95% CI 0.781–0.800), and PCT alone was 0.717 (95% CI 0.708–0.727) (*p* < 0.0001 [DeLong’s test]). The predictive accuracy of model 2 (simple bacteremia risk score) was 0.87 (95% CI 0.87–0.88) with sensitivity of 0.958, specificity of 0.215, positive predictive value of 0.900, and negative predictive value of 0.411. Using the validation cohort, an internal validation of the predictive value of PCT versus another prediction rule was performed (model 1, model 2, and PCT alone). A total of 4504 patients were enrolled and analyzed for internal validation. The bacteremia prediction performance of model 1 was 0.805 (95% CI 0.785–0.824), model 2 was 0.791 (95% CI 0.772–0.810), and that of PCT alone was 0.753 (95% CI 0.773–0.774) (*p* < 0.0001 [DeLong’s test]) (Table 4, Figure 4). The predictive accuracy of model 2 (simple bacteremia risk score) was 0.86 (95% CI 0.84–0.87) with sensitivity of 0.93, specificity of 0.310, positive predictive value of 0.905, and negative predictive value of 0.389. The constructed model calibration plot is presented in Figure 4, showing that predicted probabilities were close to the observed bacteremia. 

### 3.6. Subgroup Analysis with Missing Data Imputation 

To address missing data, traditional approaches were used by imputing missing values using the median of the observed values. After imputation of missing data, 43,294 patients were enrolled and included in the second analysis. The population was randomly divided into a derivation cohort (n = 30,305 [70% randomly selected sample]) and a validation cohort (n = 12,989 [30% randomly selected sample]). The performances of model 1, model 2, and PCT were 0.797 (95% CI 0.783–0.811), 0.778 (95% CI 0.764–0.793), and 0.706 (95% CI 0.690–0.772), respectively; DeLong’s test had a *p* < 0.0001 in the validation cohort (Supplementary Figure S1). In addition, 13,832 had normal PCT within <0.5 ng/mL among 43,294 patients. The proportion of patients with bacteremia was also higher at higher score levels, even in the subgroup with normal PCT (Supplementary Figure S2). The simple bacteremia score was 0.686 (95% CI 0.664–0.709) in the derivation cohort and 0.671 (95% CI 0.624–0.718) in the normal PCT group. 

## 4. Discussion

In this derivation and validation analysis of 22,519 patients with suspected infection, we compared the predictive performance of model 1 comprising 16 variables based on factors associated with bacteremia, model 2 comprising five variables, and PCT which was used alone. This study derived a simple bacteremia prediction score (i.e., model 2) to simplify the prediction of bacteremia, demonstrating a comparable performance to that of model 1. The simple bacteremia score (model 2) demonstrated a similar performance to that of model 1 (AUC of 0.805 vs. 0.791), whereas PCT, as the best individual variable, yielded a weaker AUC of 0.753. The risk for developing bacteremia was proportional to an increase in score. 

Strengths of the present study include its large population size with validation, risk stratification guiding blood cultures, applicability to a wide range of populations, including low-risk patients, heterogeneous characteristics of the ED, and simplicity of the score. We identified significant predictors of bacteremia in a large derivation cohort and validated the performance of the model. An increased risk for bacteremia has been an important issue among patients with sepsis; consequently, false-positive blood cultures are associated with prolonged hospital stays and increased costs, with no definitive guidelines for blood cultures [5,7]. Several studies have explored bacteremia prediction tools; however, these have been limited to specific diseases and complexity [18,19,20,21,22]. Therefore, risk stratification of bacteremia using this simple tool may help identify patients who require a blood culture. Conversely, a score of 0 can aid in the direction of not performing a blood culture because the probability of bacteremia was <2% at this score. Miquel et al. established a bacteremia rate < 8% for patients with pneumonia with a score ≤1 using six variables [23]. Potentially, the application of our simple bacteremia score (model 2) may better eliminate unnecessary blood cultures and the misuse/abuse of antibiotics. 

Moreover, the simplicity of the bacteremia prediction score makes it convenient and useful for clinicians. In a recent study, David et al. used a modified Shapiro score (MSS) ≥ 3 and NLR > 12, which demonstrated an equal ability to predict bacteremia, with AUCs of 0.71 and 0.74, respectively; however, combining MSS and NLR did not increase the predictive performance [24]. Although Chun-Yuan et al. established an AUC of 0.867 (95% CI 0.806–0.928) using a combination of four factors (age ≥ 65 years, involvement area, liver cirrhosis, systemic inflammatory response syndrome); however, this score was limited in patients with cellulitis [22]. Lars et al. reported an AUC of 0.86 (95% CI 0.83–0.89) using a combination of four biomarkers (NLR, CRP, Lac, and PCT) [25]. However, it was likely easier to distinguish bacteremia in the population, which had a relatively high risk for bacteremia because verified bacterial infection reached 55.6% of enrolled patients. However, the present study yielded an AUC of approximately 0.80 using simple variables, even in heterogeneous populations (12% bacteremia). Therefore, our simple bacteremia score would be applicable in a wide range of populations containing low-risk patients and heterogeneous ED characteristics with the advantage of simplicity. 

Regarding the risk factors for bacterial infection, the Shapiro score, which was originally developed to rule out patients with a low risk for positive blood cultures, is commonly used [14,26]. Our variables are consistent with the Shapiro scores and the previous literature. Among the variables analyzed in this study, PCT was the most influential independent predictor. Afshan et al. reported that AUCs for PCT were 0.781 and 0.70 [27] in a study by Sibtain et al. [28], outperforming CRP in both studies. Abderrahim et al. reported that a PCT threshold, ranging from ≤0.4 to ≥0.75 ng/mL, demonstrated high diagnostic accuracies for bacteremia in a cross-sectional study [29]. Marik et al. also suggested PCT < 0.5 ng/mL as an effective screening tool to exclude bacteremia, and NLR as a screening test for bacteremia when PCT is unavailable [30]. The NLR has been described as a predictor of bacteremia [14]. Ratzinger et al. found that neutrophils were the best individual variable to predict bacteremia, with an AUC of 0.694 [31]. Thrombocytopenia has also been known to be a prognostic marker for bacteremia and associated with bacteremia [32,33,34,35]. Lac, a prognostic biomarker for sepsis, is not considered to be specific for diagnosing sepsis [36]; however, several studies have shown that Lac is a biomarker for diagnosing bacterial sepsis [25]. 

Previous studies have proposed several models to predict bacteremia using not only simple predictors but also > 10 variables. In a cross-sectional study, models with 20 and 10 variables were established with AUCs of 0.767 and 0.759, respectively [31]. Paul et al. reported that a computerized decision support system (TREAT) yielded an AUC of 0.68 (95% CI 0.63–0.73) in the first cohort and 0.70 (95% CI 0.67–0.73) in the second cohort in predicting bacteremia [37]. Another study by Ratzinger et al. proposed 29 parameters to predict bacteremia, with an AUC of 0.729 (95% CI 0.679–0.779), whereas PCT exhibited an AUC similar to that reported by machine learning methods that failed to improve the moderate diagnostic accuracy of PCT [38]. 

Our study had several limitations, the first of which were its single center, cohort design. As a result, the proposed predictive model requires external validation to confirm the fitting of models. Nevertheless, this scoring algorithm enables ease of usability. Second, although we attempted to identify risk factors for bacteremia, other possible confounding factors should be considered, and significant predictors that have clinical validity should be identified. Third, in the subgroup analysis of missing imputations, most single-imputation methods provided biased estimates and incorrect standard errors. Fourth, patients taking antibiotics before their ED visits were not investigated. This could have affected the results of detecting bacteremia, although it could have made the models more practical. Fifth, this study lacks the data such as investigation of underlying disease states including diabetes mellitus or immunosuppression that may impact rates of bacteremia. 

## 5. Conclusions

In this study, we developed and validated a simple bacteremia prediction score, which using only five variables, demonstrated a similar performance to the model with sixteen variables using all laboratory results and vital signs. This simple score is useful in predicting bacteremia and assisting in clinical decisions. 

## Figures and Tables

**Table 1:** The baseline characteristics of the study population.

	Overall	No Bacteremia	Bacteremia	*p* Value
	N = 22,519	N = 19,818 (88)	N = 2701 (12)	
Age > 65	9741 (43.3)	8285 (41.8)	1456 (53.9)	<0.001
Gender, Male	12,232 (54.3)	10,771 (54.3)	1461 (54.1)	0.816
Vital Signs				<0.001
Blood pressure SBP < 90 or MAP ≤ 65	2047 (9.1)	1535 (7.7)	512 (19.0)	<0.001
HR > 130 beats/min	2557 (11.4)	2090 (10.5)	467 (17.3)	<0.001
RR ≥ 22 cycles/min	4888 (21.7)	4215 (21.3)	673 (24.9)	<0.001
BT (>38 °C or <36 °C)	10,018 (44.5)	8407 (42.4)	1611 (59.6)	<0.001
Laboratories				
WBC (>12,000 or <4000/mm^3^)	11,696 (51.9)	10,136 (51.1)	1560 (57.8)	<0.001
PLT < 150,000/mm^3^	609 (2.7)	455 (2.3)	154 (5.7)	<0.001
Band Neutrophil > 5%	578 (2.6)	434 (2.2)	144 (5.3)	<0.001
ANC < 1.5 or >8.3	12,563 (55.8)	10,851 (54.8)	1712 (63.4)	<0.001
ALC > 2.9 or <0.9	17,470 (77.6)	15,042 (75.9)	2428 (89.9)	<0.001
Neutrophil–Lymphocyte Ratio	6.9 (3.3–12.9)	6.4 (3.1–11.7)	12.4 (5.9–22.3)	<0.001
Albumin, g/dL	3.7 (3.2–4.1)	3.7 (3.2–4.1)	3.5 (2.9–3.9)	<0.001
Creatinine, mg/dL	0.9 (0.7–1.2)	0.8 (0.7–1.2)	1.1 (0.8–1.7)	<0.001
Lactate, mmol/L	1.7 (1.2–2.5)	1.6 (1.2–2.3)	2.4 (1.6–3.8)	<0.001
CRP, mg/dL	7.5 (2.7–14.9)	7.0 (2.5–14.0)	11.3 (4.8–19.8)	<0.001
Procalcitonin, mg/dL	0.3 (0.1–4.5)	0.2 (0.1- 0.8)	2.6 (0.6–14.2)	<0.001
NLR ≥ 10	7793 (34.6)	6191 (31.2)	1602 (59.3)	<0.001
Lactate > 2 mmol/L	8439 (37.5)	6784 (34.2)	1655 (61.3)	<0.001
Creatinine > 1.5 mg/dL	3881 (17.2)	3074 (15.5)	807 (29.9)	<0.001
Albumin < 3.5 g/dL	8396 (37.3)	7074 (35.7)	1322 (48.9)	<0.001
CRP > 8 mg/dL	10,734 (47.7)	9045 (45.6)	1689 (62.5)	<0.001
PCT > 0.5 mg/dL	8687 (38.6)	6608 (33.3)	2079 (77.0)	<0.001
Vasopressor use	2504 (11.1)	1673 (8.4)	831 (30.8)	<0.001

The data are presented as mean ± standard deviation, median (IQR) or number (%). Abbreviations: IQR, interquartile range; SBP, systolic blood pressure; MAP, mean arterial pressure; HR: heart rate; RR, respiratory rate; BT, body temperature; WBC, white blood cell; PLT, platelet; ANC, absolute neutrophil count; ALC, absolute lymphocyte count; NLR, neutrophil–lymphocyte ratio; CRP, c-reactive protein; PCT, procalcitonin.

**Table 2:** Multivariable logistic regression of top five predictors of bacteremia in all variables, derivation cohort, and validation cohort.

Multivariable	(Total)			(Derivation)			(Validation)		
Predictor Set	OR	95% CI	*p*	OR	95% CI	*p*	OR	95% CI	*p*
PCT > 0.5 mg/dL	4.78	4.33–5.29	<0.001	4.65	4.16–5.20	<0.001	5.33	4.28–6.69	<0.001
NLR ≥ 10	2.25	2.05–2.46	<0.001	2.27	2.05–2.51	<0.001	2.19	1.80–2.67	<0.001
Lactate > 2 mmol/L	1.97	1.80–2.15	<0.001	2.00	1.81–2.20	<0.001	1.87	1.54–2.28	<0.001
PLT < 150,000/mm^3^	2.64	2.14–3.25	<0.001	2.72	2.15–3.43	<0.001	2.37	1.48–3.72	<0.001
BT (>38 °C or <36 °C)	2.06	1.89–2.25	0.854	2.04	1.85–2.25	<0.001	2.16	1.78–2.62	<0.001

The *p* value of goodness-of-fit with the Hosmer–Lemeshow test = 0.131. Abbreviations: OR, odds ratio; CI, confidence interval; PCT, procalcitonin; NLR, neutrophil–lymphocyte ratio; PLT, platelet; BT, body temperature.

**Table 3:** Clinical prediction scale (simple bacteremia risk score of the final model).

Risk Factors	Points (Score)
Procalcitonin > 0.5 mg/dL	2
Neutrophil–Lymphocyte Ratio ≥ 10	1
Lactate > 2 mmol/L	1
Platelet < 150,000/mm^3^	1
Body Temperature (>38 °C or <36 °C)	1

**Table 4:** Area under the receiver operating characteristics curve (AUC) of model 1, simple score, and procalcitonin to predict bacteremia in the derivation and validation data.

Predictor Set	Derivation	Validation
Model 1 (All 16)	0.803 (0.794–0.813)	0.805 (0.785–0.824)
Model 2 (Simple score)	0.790 (0.781–0.800)	0.791 (0.772–0.810)
Procalcitonin	0.717 (0.708–0.727)	0.753 (0.733–0.774)

Model 1: bacteremia predicting model based on 16 predictors associated with bacteremia. Model 2: simple bacteremia score using top five predictors associated with bacteremia among sixteen variables.

## Data Availability

The datasets used in this work are available upon reasonable request from the corresponding author and are not publicly available.

## Supplementary Materials

The following supporting information can be downloaded at: https://www.mdpi.com/article/10.3390/jpm14010057/s1. Figure S1: Receiver operating characteristic (ROC) curves of all models, simple score, and procalcitonin to predict bacteremia in the validation data with missing data imputation; Figure S2: Distribution of bacteremia by simple bacteremia score levels in normal procalcitonin level of <0.5; Table S1: Baseline characteristics of the derivation and validation cohort; Table S2: Univariable logistic regression in all 16 variables; Table S3: Multivariable logistic regression in all 16 variables.
