# Supplementary material for: A Simple Bacteremia Score for Predicting Bacteremia in Patients with Suspected Infection in the Emergency Department: A Cohort Study
Source: J Pers Med. 2023 Dec 30;14(1):57. doi: 10.3390/jpm14010057 (PMC10817606; doi:10.3390/jpm14010057)
Supplement: Supplementary file 1 [file jpm-14-00057-s001.zip › jpm-2753022-supplementary.pdf]

Supplementary file

Supplementary Table S1. baseline characteristics of derivation and validation cohort

	Derivation cohort (n = 18015)	Validation cohort (n = 4504)
Age>65	7775 (43.2)	1966 (43.7)
Gender	9747 (54.1)	2485 (55.2)
Vital Sign		
SBP, mm Hg SBP<90 or MAP≤65	1604 (8.9)	443 (9.8)
HR >130 beats/min	2073 (11.5)	484 (10.8)
RR ≥22 cycles/min	3871 (21.5)	1017 (22.6)
BT>38°C or <36°C	8017 (44.5)	2001 (44.4)
Laboratories		
WBC, /mm3 >12000 or <4000	9354 (51.9)	2342 (52)
PLT < 150000/mm^3	482 (2.7)	127 (2.8)
Band-Neutrophil>5%	447 (2.5)	131 (2.9)
ANC < 1.5 or > 8.3	10043 (55.8)	2520 (56.0)
ALC > 2.9 or < 0.9	13978 (77.6)	3492 (77.5)
NLR	6.9 (3.3-12.9)	6.7 (3.2-12.8)
Albumin, g/dL	3.7 (3.2-4.1)	3.7 (3.2-4.1)
Creatinine, mg/dL	0.9 (0.7-1.2)	0.9 (0.7-1.3)
Lactate, mmol/L	1.7 (1.2-2.5)	1.7 (1.2-2.5)
CRP, mg/dL	7.5 (2.7-14.8)	7.5 (2.8-15.1)
Procalcitonin, mg/dL	0.3 (0.1-1.3)	0.3 (0.1-1.3)
NLR ≥10	6247 (34.7)	1546 (34.3)
Lactate >2 mmol/L	6729 (37.4)	1710 (38.0)
Creatinine > 1.5 mg/dL	3082 (17.1)	799 (17.7)
Albumin < 3.5 g/dL	6665 (37)	1731 (38.4)
CRP > 8 mg/dL	8570 (47.6)	2164 (48.1)
PCT > 0.5 mg/dL	6894 (38.3)	1793 (39.8)
Vasopressor use	1974 (11.0)	530 (11.8)
Bacteremia	2145 (11.9)	556 (12.3)
Gram positive bacteremia	491 (2.7)	132 (2.9)
Gram negative bacteremia	1551 (8.6)	390 (8.7)

The data are presented as mean±standard deviation, median (IQR) or number (%).

Abbreviations: IQR, interquartile range; SBP, systolic blood pressure; MAP, mean arterial pressure; HR: heart rate; RR, respiratory rate; BT, body temperature; WBC, white blood cell; PLT, platelet; ANC, absolute neutrophil count; ALC, absolute lymphocyte count; NLR, neutrophil-lymphocyte ratio; CRP, c-reactive protein; PCT, procalcitonin.

Supplementary Table S2. Univariable logistic regression in all 16 variables

Predictor set	Univariable (Total)			(Derivation)			(validation)		
	OR	95%CI	p	OR	95%CI	p	OR	95%CI	p
PCT > 0.5 mg/dL	3.21	2.96- 3.48	<0.001	3.25	2.96- 3.56	<0.001	3.06	2.56- 3.67	<0.001
NLR ≥10	3.04	2.80- 3.30	<0.001	3.06	2.79- 3.36	<0.001	2.96	2.47- 3.56	<0.001
Lactate >2 mmol/L	2.32	2.12- 2.54	<0.001	2.3	2.08- 2.55	<0.001	2.4	1.96- 2.92	<0.001
Creatinine > 1.5 mg/dL	1.73	1.59- 1.87	<0.001	1.72	1.57- 1.88	<0.001	1.75	1.46- 2.09	<0.001
Albumin < 3.5 g/dL	1.63	1.50- 1.76	<0.001	1.64	1.50- 1.79	<0.001	1.59	1.33- 1.90	<0.001
Age>65	1.23	1.12- 1.35	<0.001	1.17	1.05- 1.30	0.004	1.46	1.19- 1.77	<0.001
RR ≥22 cycles/min	2.01	1.85- 2.18	<0.001	2.01	1.84- 2.21	<0.001	1.97	1.65- 2.37	<0.001
BT>38°C or <36°C	2.79	2.50- 3.11	<0.001	2.78	2.46- 3.14	<0.001	2.8	2.20- 3.53	<0.001
SBP<90 or MAP≤65	1.77	1.59- 1.98	<0.001	1.82	1.61- 2.06	<0.001	1.58	1.22- 2.03	<0.001
HR >130 beats/min	6.68	6.08- 7.35	<0.001	6.6	5.95- 7.34	<0.001	7.02	5.69- 8.72	<0.001
CRP > 8 mg/dL	1.99	1.83- 2.16	<0.001	1.93	1.76- 2.12	<0.001	2.22	1.85- 2.68	<0.001
ALC > 2.9 or < 0.9	2.82	2.49- 3.22	<0.001	2.86	2.48- 3.32	<0.001	2.68	2.04- 3.58	<0.001
PLT < 150000/mm^3	2.57	2.13- 3.10	<0.001	2.6	2.10- 3.20	<0.001	2.48	1.62- 3.69	<0.001
ANC < 1.5 or > 8.3	1.43	1.32- 1.55	<0.001	1.38	1.26- 1.51	<0.001	1.65	1.37- 1.99	
WBC, /mm3 >12000 or <4000	1.31	1.20- 1.42	<0.001	1.3	1.19- 1.42	<0.001	1.34	1.12- 1.60	0.002
Band-Neutrophil>5%	2.52	2.07- 3.04	<0.001	2.55	2.04- 3.17	<0.001	2.37	1.56- 3.53	<0.001

Abbreviations: OR, odds ratio; CI, confidence interval; PCT, procalcitonin NLR, neutrophil-lymphocyte ratio; RR, respiratory rate; BT, body temperature; SBP, systolic blood pressure; MAP, mean arterial pressure; HR: heart rate; CRP, c-reactive protein; ALC, absolute lymphocyte count; PLT, platelet; ANC, absolute neutrophil count; WBC, white blood cell.

Supplementary Table S3. Multivariable logistic regression in all 16 variables

Predictor set	Multivariable (Total)			(Derivation)			(Validation)		
	OR	95%CI	p	OR	95%CI	p	OR	95%CI	p
PCT > 0.5 mg/dL	3.99	3.58-4.43	<0.001	3.92	3.49-4.42	<0.001	4.25	3.35-5.40	0.0000
NLR ≥10	2.04	1.85-2.25	<0.001	2.09	1.87-2.33	<0.001	1.90	1.53-2.37	0.0000
Lactate >2 mmol/L	1.87	1.71-2.06	<0.001	1.91	1.72-2.12	<0.001	1.75	1.42-2.16	0.0000
Creatinine > 1.5 mg/dL	1.27	1.14-1.40	<0.001	1.25	1.11-1.40	0.0002	1.33	1.05-1.67	0.0161
Albumin < 3.5 g/dL	1.01	0.92-1.11	0.854	1.02	0.92-1.14	0.721	0.97	0.79-1.20	0.778
Age>65	1.41	1.29-1.54	<0.001	1.43	1.29-1.58	<0.001	1.33	1.08-1.62	0.0058

RR ≥22 cycles/min	0.85	0.76-0.94	0.002	0.80	0.71-0.90	<0.001	1.04	0.83-1.31	0.706
BT>38°C or <36°C	2.18	1.99-2.39	<0.001	2.14	1.93-2.37	<0.001	2.36	1.92-2.90	<0.001
SBP<90 or MAP≤65	1.54	1.36-1.75	<0.001	1.49	1.30-1.72	<0.001	1.74	1.32-2.28	<0.001
HR >130 beats/min	1.16	1.02-1.31	0.023	1.18	1.03-1.36	0.020	1.09	0.81-1.46	0.585
CRP > 8 mg/dL	1.23	1.12-1.36	<0.001	1.19	1.07-1.33	0.001	1.41	1.14-1.74	0.00
ALC > 2.9 or < 0.9	1.60	1.38-1.84	<0.001	1.60	1.36-1.88	<0.001	1.59	1.16-2.17	0.004
PLT < 150000/mm^3	2.50	2.01-3.10	<0.001	2.60	2.04-3.31	<0.001	2.19	1.36-3.52	0.001
ANC < 1.5 or > 8.3	0.92	0.81-1.03	0.156	0.86	0.75-0.99	0.030	1.17	0.90-1.52	0.253
WBC, /mm3 >12000 or <4000	0.96	0.86-1.08	0.490	0.99	0.87-1.13	0.911	0.84	0.66-1.09	0.187
Band-Neutrophil>5%	1.14	0.92-1.41	0.244	1.13	0.88-1.43	0.340	1.16	0.73-1.84	0.536

Abbreviations: OR, odds ratio; CI, confidence interval; PCT, procalcitonin NLR, neutrophil-lymphocyte ratio; RR, respiratory rate; BT, body temperature; SBP, systolic blood pressure; MAP, mean arterial pressure; HR: heart rate; CRP, c-reactive protein; ALC, absolute lymphocyte count; PLT, platelet; ANC, absolute neutrophil count; WBC, white blood cell.

Figure S1. Receiver operating characteristic (ROC) curves of all models, simple score and procalcitonin to predict bacteremia in the validation data with missing data imputation

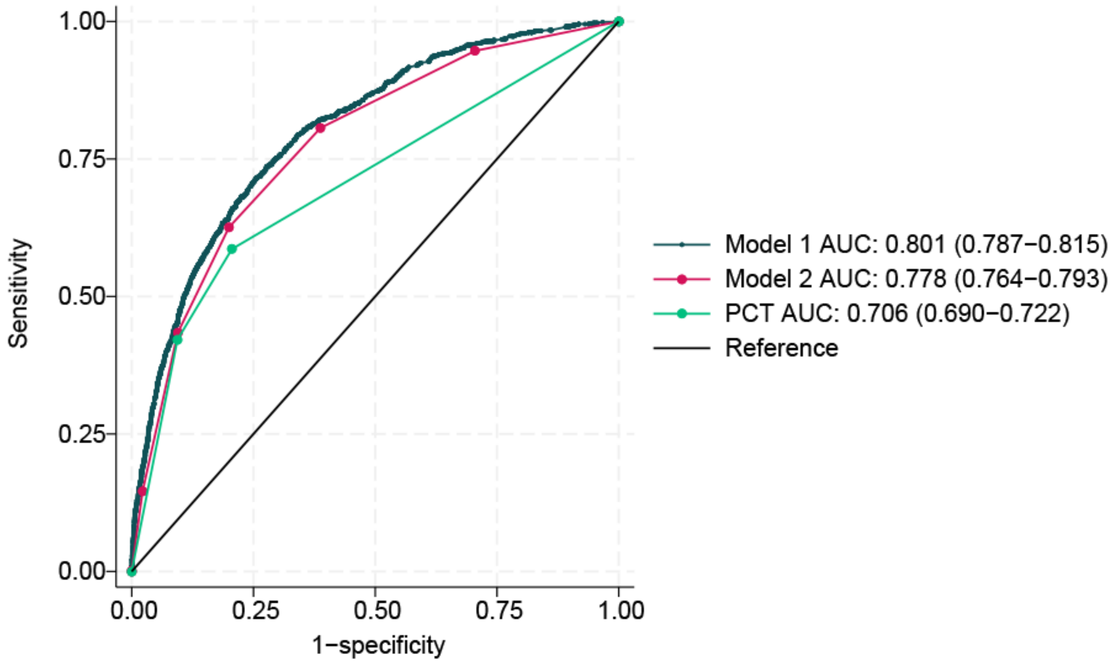

Figure S2. Distribution of bacteremia by simple bacteremia score levels in normal procalcitonin level of <0.5

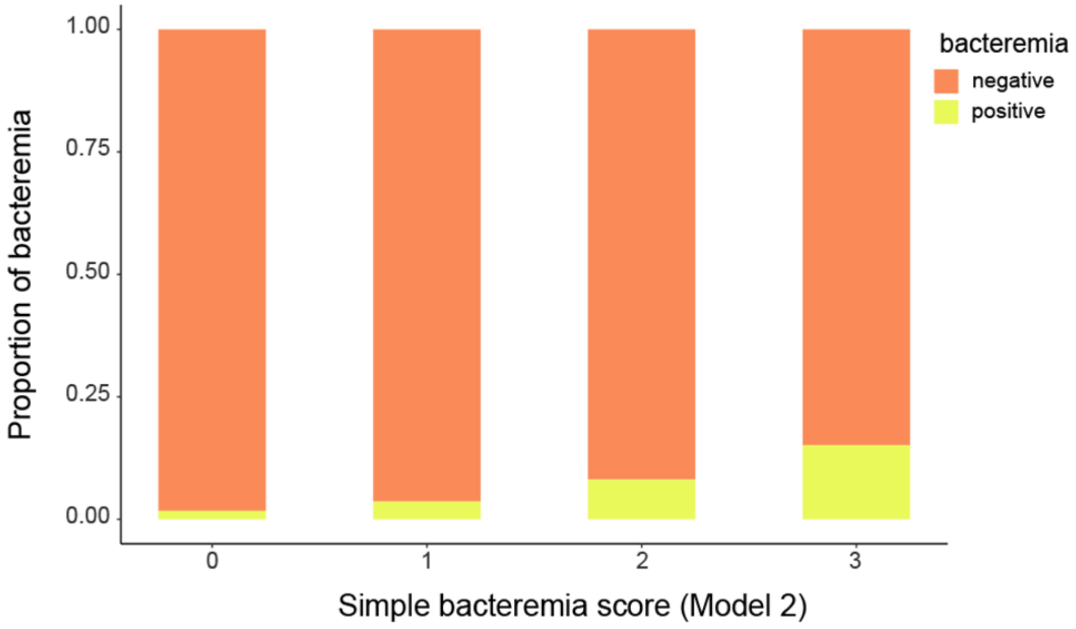

1.7% 3.6% 8.1% 15.1%
